# Global Rhes knockout in the Q175 Huntington’s disease mouse model

**DOI:** 10.1371/journal.pone.0258486

**Published:** 2021-10-14

**Authors:** Taneli Heikkinen, Timo Bragge, Juha Kuosmanen, Teija Parkkari, Sanna Gustafsson, Mei Kwan, Jose Beltran, Afshin Ghavami, Srinivasa Subramaniam, Neelam Shahani, Uri Nimrod Ramírez-Jarquín, Larry Park, Ignacio Muñoz-Sanjuán, Deanna M. Marchionini

**Affiliations:** 1 Charles River Discovery Services, Kuopio, Finland; 2 Psychogenics, Paramus, New Jersey, United States of America; 3 The Scripps Research Institute, Department of Neuroscience, Jupiter, Florida, United States of America; 4 CHDI Management/CHDI Foundation, New York, New York, United States of America; Louisiana State University Health, Shreveport, UNITED STATES

## Abstract

Huntington’s disease (HD) results from an expansion mutation in the polyglutamine tract in huntingtin. Although huntingtin is ubiquitously expressed in the body, the striatum suffers the most severe pathology. Rhes is a Ras-related small GTP-binding protein highly expressed in the striatum that has been reported to modulate mTOR and sumoylation of mutant huntingtin to alter HD mouse model pathogenesis. Reports have varied on whether Rhes reduction is desirable for HD. Here we characterize multiple behavioral and molecular endpoints in the Q175 HD mouse model with genetic Rhes knockout (KO). Genetic RhesKO in the Q175 female mouse resulted in both subtle attenuation of Q175 phenotypic features, and detrimental effects on other kinematic features. The Q175 females exhibited measurable pathogenic deficits, as measured by MRI, MRS and DARPP32, however, RhesKO had no effect on these readouts. Additionally, RhesKO in Q175 mixed gender mice deficits did not affect mTOR signaling, autophagy or mutant huntingtin levels. We conclude that global RhesKO does not substantially ameliorate or exacerbate HD mouse phenotypes in Q175 mice.

## Introduction

Huntington’s disease (HD) is an autosomal dominant neurodegenerative disorder caused by a CAG trinucleotide repeat expansion in the huntingtin (*HTT)* gene, which encodes an expanded polyglutamine tract in the huntingtin (HTT) protein [1,2]. The striatum is one of the earliest and most severely affected brain structures in HD [3–5].

Ras homolog enriched in striatum (Rhes) is a Ras-related small GTP-binding protein highly expressed in the striatum [6] that modulates activation of G proteins, to which GPCRs are coupled [7]. Rhes mRNA and protein is reduced in postmortem caudate and striatum of HD mouse models [8–10] (HDinHD: www.hdinhd.org). Mechanistically, Rhes acts through at least 2 mechanisms, sumoylation of mutant (m)HTT and mTOR modulation. *In vitro* studies suggest that Rhes acts like a SUMO E3-like ligase and interacts with mHTT to promote its SUMOylation that is linked to cellular toxicity [11–14]. Rhes mediated toxicity has been demonstrated in various cellular HD models [15–19]. SUMOylation of mHTT decreases aggregated mHTT and increases soluble mHTT *in vitro* [11,12]. The mechanisms or downstream effector(s) of Rhes/mHTT-mediated cells death remains unknown. Rhes can bind and activate mTOR in cell culture through increased phosphorylation of S6K, S6RP and 4EBP1, and can promote L-DOPA-mediated mTORC1 activation in the striatum of hemiparkinsonian mice [20,21]. Rhes resembles Rheb, a known activator of mTOR [22] and in a cell culture model mHTT can promote nutrient-induced mTORC1 activity via Rheb [23], raising the possibility that dysregulation of striatal mTORC1 signaling via Rhes and Rheb could affect HD [24].

Despite cellular models supporting the toxic role of Rhes, its desired pharmacology in HD remains unclear since prior studies in HD rodent models report both protective and detrimental effects of Rhes on HD-like behavioral and anatomical deficits [9,10,25–28].

Our goal in this study was to thoroughly characterize multiple behavioral and molecular endpoints in a genetic Rhes knockout (KO) of the well-characterized Q175 HD mouse model. We show that genetic KO of Rhes does not greatly modulate fine motor behaviors or volumetry, metabolic or DARPP32 deficits that are usually seen in the Q175 mouse. Furthermore, there was no effect on mTOR signaling, autophagy or mHTT levels with genetic RhesKO in the Q175 model.

## Materials and methods

### Animals

All animal experiments were performed as specified in the licence authorised by the National Animal Experiment Board of Finland and according to the National Institutes of Health (Bethesda, MD, USA) guidelines for the care and use of laboratory animals and humane endpoints were used. Animals were monitored daily by laboratory personnel; the animals’ welfare was assessed by observing the following signs: general appearance (dehydration, weight loss, abnormal posture, condition of skin and fur, signs of pain); ambulation, behavior and clinical signs (eating, drinking, urinating, defecating). The zQ175 knock in (KI) neo- (Q175; C57BL/6J background) [29] and Rhes knock-out (RhesKO; C57BL/6J background; [30] mice were bred to generate experimental mice. The average CAG repeat size of the Q175^+/-^ mice was 186. Mice were randomized into groups so that whole litters of mice did not end up in a single testing group. Mice were housed in groups of 4 and separated by gender. In each cage, one WT mouse was included. Mice acclimated to the experimental room for at least one hour prior to the beginning of any experiment. Experimentation was conducted in a blinded manner. There was a single cohort of female mice that was used for behavior, neuroimaging, mHTT and autophagy quantification; mice were humanely euthanized with pentobarbital and checked for no response to a tail or toe pinch prior to decapitation. A second cohort of mixed gender was used to look at mTOR signaling and DARPP32; mice were humanely euthanized by focused beam microwave irradiation, which results in death in <1 sec. Briefly mice were restrained with their head positioned evenly, the holder was then placed in the microwave chamber and each mouse was euthanized via focused microwave irradiation (5kW) for 0.95sec, for striatal fixation. Following fixation, mice were placed in a plastic bag and submerged for 10 minutes to cool prior to dissection.

### Body weight

Body weight was measured once a week from 21–54 weeks of age.

### Motor function

#### Fine motor skill and gait analysis

Fine motor skills and gait of the mice were evaluated using an apparatus (Motorater, TSE-systems GmbH, Bad Homburg, Germany) designed for the assessment of fine motor skills in rodents. The equipment consists of a brightly illuminated plexiglas corridor (153 x 5 x 10 cm) under which is situated a high-speed camera. A few days before the test sessions, under light isoflurane anesthesia, the fur of the limbs was removed. On the day of testing, the mice were marked in appropriate points of body, such as joints of limbs and parts of tail to ease the data analysis process. The performance of the mice was assessed during walking along the corridor and was recorded with a high-speed video-camera (300 fps). Approximately 5–6 complete strides were analyzed from each mouse. Only strides with continuous ambulatory movement were included in the data. Using a specific setup of mirrors, the performance of the mouse can be detected simultaneously from both sides and the underside. The movement of the mice was analyzed from the three views, first using the Simi Reality Motion Systems (Unterschleissheim, Germany) and the obtained raw marker trajectory data (trajectory of each marked point of body during movement) was further analyzed by a custom analysis system. Altogether, 24 different points of body were tracked from each mouse. Data were analyzed for distinctive kinematic parameters using custom made analysis software, followed by principal component (PC) analysis. The Overall Gait Score was based on the differences between Q175 and WT groups in all the PC scores. First, a kinematic “fingerprint”, combination of original variables which characterize the Q175 with respect to WT, was identified. The “fingerprint”, or discriminant vector, indicated the contribution of each individual parameter to the Overall Gait Score. The scores were obtained by projecting the (normalized) parameter data of each individual mouse onto the discriminant vector. Details of the fine motor kinematic analysis have been reported previously [31].

### Magnetic resonance imaging and spectroscopy

MRI acquisitions were performed at 6 and 12 months of age using a horizontal 11.7T magnet with bore size 160 mm equipped with a gradient set capable of max. gradient strength 750 mT/m and interfaced to a Bruker Avance III console (Bruker Biospin GmbH, Ettlingen, Germany). A volume coil (Bruker Biospin GmbH, Ettlingen, Germany) was used for transmission and a surface two-element array coil for receiving (Rapid Biomedical GmbH, Rimpar, Germany). Isoflurane-anesthetized mice were fixed to a head holder and positioned in the magnet bore in a standard orientation relative to gradient coils.

Structural MRI was performed with a standard Turbo-RARE sequence with TEeff of 34 ms (RARE factor of 8), TR of 2500 ms and 8 averages. Thirty-one 0.45 mm slices were collected with field-of-view of 20x20 mm^2^ and 256x256 matrix (78 microns in-plane resolution). Region of interest analysis was performed in MATLAB (Mathworks Inc., Natick, MA, USA) environment observer blinded for study groups. Whole brain, striatum and cortex volumes were analyzed.

Proton (^1^H) MR spectroscopy data were collected using the same experimental setup. For the acquisition, right striatal voxel (1.8x1.8x2.0 mm^3^, 6.5 μl localized volume, Q175 KI mice) was selected based on structural MRI described above. Automatic 3D gradient echo shimming algorithm was used to adjust B0 homogeneity in the voxel. The water signal was suppressed using variable power RF pulses with optimized relaxation delays (VAPOR) to obtain B1 and T1 insensitivity. A PRESS sequence (TE = 10 ms) combined with outer volume suppression (OVS) was used for the pre-localization. Data were collected by averaging 512 excitations (frequency corrected for each FID) with TR of 2 s, number of points 2048 and spectral width of 5 kHz. Excitation frequency was shifted -2 ppm, to minimize the chemical shift phenomenon within the selected voxel. In addition, a reference spectrum without water suppression (NT = 8) was collected from the identical voxel using the same acquisition parameters. Peak areas for resolved metabolites were analyzed using LCModel (Stephen Provencher Inc., Oakville, Canada) using >CRLB 20% as exclusion criterium for individual metabolites within analyzed spectrum.

### qPCR

The qPCR assay was designed to amplify exons 1 and 2 with the hydrolysis probe located in exon 2 of the endogenous Rhes transcript; this assay should not amplify in Rhes KO due to incorporation of the IRES-EGFP and PGKneo insertion. Hemisection of striatal tissue from WT and Rhes KO was used to validate this assay. RNA and cDNA was prepared from the fresh frozen tissue samples and qPCR was performed with the following primers and probe set: Forward-5’-CACCTCCAGGAGCTTCCA-3’; reverse-5’AGTTCCCACTGGACAAGGTC-3’; probe-Universal Probe Library Single Probe Set#21 (Roche Applied Sciences, cat# 04686942001).

### Western blotting

Striatum tissues were sonicated 30sec with probe sonicator in 1% SDS at 90°-95°C, and then incubated on wet ice for 30min. Sonicated lysate was centrifuged for 10min at 10,000 rpm. Supernatants were collected and protein amount determined by using Bio-Rad DC protein quantification assay. Protein titration curves were run to determine optimal protein loading for each target. Protein samples were denatured in Laemmli buffer (Bio-Rad)/2-Mercapthoethanol (Sigma-Aldrich) for 5 minutes at 95°C. Denatured protein samples were separated on 4–12% SDS-PAGE Criterion Gels (Bio-Rad). After electrophoresis, proteins were transferred from gel to Hybond-LFP PVDF membranes (GE Healthcare Bioscience) by electroblotting. Non-specific binding of antibodies was blocked with 5% w/v BSA in 1X TBST for one hour. After a brief rinse in TBST, the blots were probed with the following primary antibodies: Rhes (gift from Srini Subramaniam), DARPP32 (Abcam), pS6^S235/236^, p-mTOR^S2448^, p-4EB-P1^S65^, S6RP, mTOR, 4EB-P1, GAPDH (Cell signaling technology) or vinculin (EMD Millipore). Primary antibodies were each prepared with 1% w/v BSA in 1x TBST at 4°C overnight. Protein-PVDF blots were washed once for 15 minutes followed by 3 more 5 minutes washes with TBST. Protein-PVDF blots were then incubated with the appropriate secondary antibody prepared with 1% milk in TBST for 1 hour at room temperature. Protein-PVDF blots were washed once for 15 minutes followed by one more wash for 5 minutes. Antibody binding was detected using the ECL Plus Western Chemifluorescence Detection Kit (ThermoFisher Scientific). The detection solution was made fresh according to manufacturer’s directions and dispensed onto membranes. After 5 minutes incubation, the protein-PDVF membranes were scanned using Typhoon scanner (GE Healthcare Bioscience) using 457nm blue laser for excitation and 520nm emission filter at 400V. The scanned images from the Typhoon were analyzed with ImageQuantTL software version 7.0 (GE Healthcare Bioscience, Piscataway, NJ). Band intensities were determined using the Rolling Ball method. Final data are presented as protein normalized to housekeeping protein.

### TR-FRET and MSD

Tissue samples were weighed, transferred to a Wheaton tissue-manual homogenizer and 1:10 w/v ice cold homogenization buffer (0.4% (v/v) TritonX-100, 138 mM NaCl, 2.7 mM KCl, 8 mM Na_2_HPO_4_, 1.5 mM KH_2_PO_4_, protease inhibitors cocktail complete ultra; Roche #05892970, phosphatase inhibitors cocktail PhosSTOP; Roche #04906837001) were added. Tissue was homogenized with 20 strokes. Crude homogenate was transferred into Eppendorf tubes and frozen for 1h or longer at -80°C.

TR-FRET assays were performed transferring a proper amount of cell lysate to a low volume 384 well plate (Greiner, 784080) and adding antibody pairs diluted in lysis buffer [32]. The following antibodies were used, with Tb and D2 labeling at Cisbio (Bagnols, France): anti-LC3II-Tb (Cell Signaling; #2343), anti-LC3II-D2 (Sigma; #L7543) for the LC3II assay, anti-p62-Tb (Abnova; H00008878-M01) and anti-p62-D2 (Sigma; P0067) for the p62 assay. 5 μL of crude lysate was transferred per well to 384 low volume plates and 1 μL of antibody-mix per well was added. Plate readouts were measured using the EnVision Reader (PerkinElmer) following excitation at 320 nm, values were collected as the ratio between fluorescence emission at 665 nm and 615 nm to calculate raw data. Raw data were used for further calculation of the specific TR-FRET signal expressed as a percentage of ΔF% (DF%), and calculated as follows: ΔF% = (Signal S − Background B/ Background B) *100 and ΔF% = [(raw data sample − raw data blank)/raw data blank] * 100. Lysates were tested in technical triplicates.

For HTT quantification MSD 384-well plates (Meso Scale Discovery) were coated with capture antibody overnight 4°C and washed 3x in wash buffer (0.2% Tween-20 in PBS). Wells were blocked with blocking buffer (2% probumin/0.2% Tween-20 in PBS) for 1h at RT with rotational shaking. A total volume of 10 μL homogenate (clear lysate, diluted 1: 1 in blocking buffer) was transferred to the coated MSD plate, plates are sealed and incubated for 1h at RT with shaking at 350 rpm. After washing (3x with washing buffer), the secondary antibody/detection antibody was applied and the plates were incubated 1h at RT with shaking at 350 rpm. After final washing (3x with washing buffer), the MSD read buffer was added and the plate readout measurement was performed using the MSD reader (MESO Scale Discovery SI6000 Plate Reader). The following antibody combinations were used: For detection of expanded mutant HTT [33,34], mouse monoclonal antibody CHDI-90000830 (2B7; binding to the N-terminus of human HTT (amino acids 7–13)) as coating antibody was used in combination with mouse monoclonal antibody CHDI-90000895 (MW1; binding to the polyQ stretch of HTT) as detection antibody with a SULFO-TAG (ST) label. For detection of aggregated mutant HTT [35], mouse monoclonal antibody CHDI-90000942 (MW8; generated by using soluble human GST-tagged exon 1-Q67 boosted with exon 1-Q67 aggregates; the epitope was mapped to amino acids 83–90 (AEEPLHRP) at the C-terminus of human exon 1 HTT) as coating antibody was used in combination with mouse monoclonal antibody CHDI-90000833 (4C9; binding against the poly-proline domain of human HTT (amino acids 51–71)) as detection antibody with a SULFO-TAG (ST) label.

For detection of total mouse HTT [36], rabbit polyclonal antibody CHDI-90000147 (pAb147; binding to amino acids 37–53 within the poly-proline domain of mouse HTT) as coating antibody was used in combination with mouse monoclonal antibody MAB2166-4C8 (Millipore, with the epitope mapped to amino acids 445–459 of the human HTT] and as secondary detection antibody anti-mouse antibody (goat) SULFO-TAG labeled (Meso Scale Discovery). SULFO-TAG (ST) labeling of antibodies 4C9, MW1, and MW8 was performed using the MSD SULFO-TAG NHS-Ester reagent (Meso Scale Discovery). Lysates were tested in technical duplicates.

p/total S6RP was performed at the final concentration of 0.4 mg/ml according to the manufacturer’s instructions (MESO Scale Discovery). Lysates were tested in technical duplicates.

### Statistical analysis

All values are presented as mean ± standard error of mean (SEM), unless mentioned otherwise and differences were considered statistically significant at the p < 0.05 level. Kinematic analysis data were analyzed using two-way RM ANOVA with the Geisser-Greenhouse correction, followed by Tukeys’s test. Neuroimaging data was analyzed using statistical models with separate factors for Q175 genotype, RhesKO, and age (if multiple time points), and every two-way interaction of these three factors (fixed effects). All time series data were modeled using linear mixed effects models, subject ID set as random effect, followed by analysis of deviance (ANODE, Type III Wald chisquare tests). The post-hoc comparisons were performed using Tukey’s test on estimated marginal means of the fitted models. Data with only a single time point were analyzed using ordinary two-way ANOVA followed by Tukey’s test. These analyses were performed in R environment by making use of *lme4* and *emmeans* packages.Data from DARPP32 western blot, TR-FRET, and MSD were analyzed using one-way ANOVA followed by Bonferroni’s multiple comparison test. Data from the Rhes, RasGRP1 and Rheb Western blot was analyzed using a Student’s t-test. No outlier data was discarded.

## Results

### Confirmation of mouse genotypes

Rhes knock out was generated via insertion of a stop codon along with an EGFP cassette and an inverted neomycin cassette, downstream of the ATG in exon 2 [30]. The strategy of the qPCR assay is that primers in exons 1 and 2 with taqman probe located just downstream of ATG will only amplify in intact Rhes transcript and not in the knockout allele due to the exogenous sequence insertion. Amplification curves of WT and RhesKO striatal cDNA prepared from fresh frozen tissues showed that Rhes E1-2 qPCR assay only amplified in WT and not RhesKO (S1A Fig). ATP5B qPCR assay showed that cDNA samples were successfully prepared from RNA (S1B Fig). Western blotting for the Rhes protein confirmed that Rhes was not expressed in the RhesKO or Q175;RhesKO striatum (S1C Fig).

### Body weight

**Fig 1:**
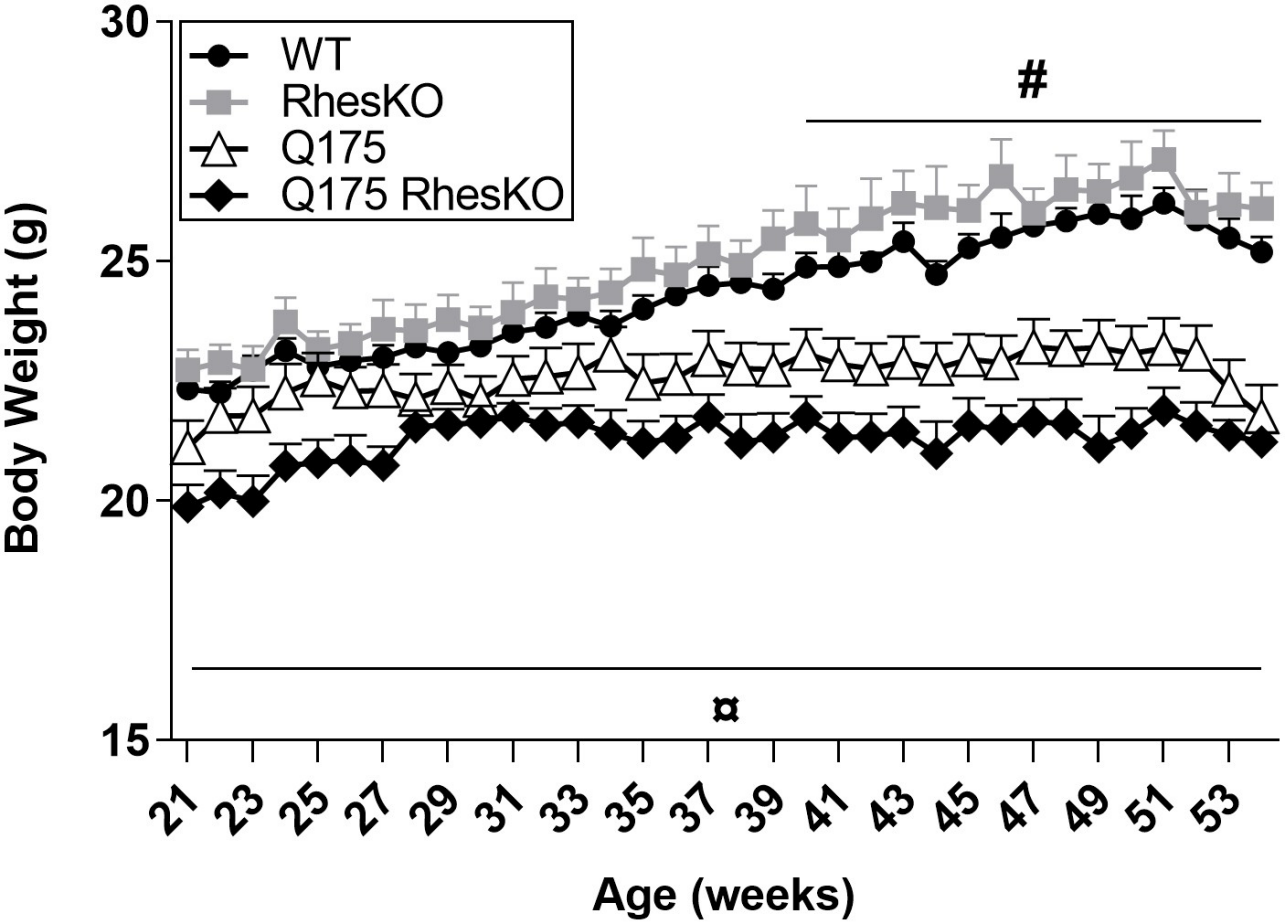
Body weight of Q175;RhesKO female mice from 21 to 54 weeks of age. Data are presented as mean ± SEM (WT n = 9; RhesKO n = 11; Q175 n = 9; Q175;RhesKO n = 9); Two-way mixed ANODE followed by Tukey’s test, # p < 0.05, Q175 vs. WT; ¤ p < 0.05, Q175;RhesKO vs. WT). There was no significant difference between Q175 and Q175;RhesKO.

There was a statistically significant interaction between the effects of Q175 and Rhes genotypes in body weight (two-way mixed ANODE, p = 0.037; Fig 1). Simple main effects analysis showed significant difference for Q175 genotype (p = 0.012), but not for Rhes (p = 0.335). Both Q175 groups had decreased body weight, compared to WT mice, from 40 weeks of age (two-way mixed ANODE followed by Tukey’s test; p < 0.05; Fig 1). Q175;RhesKO mice had decreased body weight compared to RhesKO mice and to WT mice from 21 weeks of age (p < 0.05; Fig 1).

### Motor function

#### Fine motor skill and gait analysis

**Fig 2:**
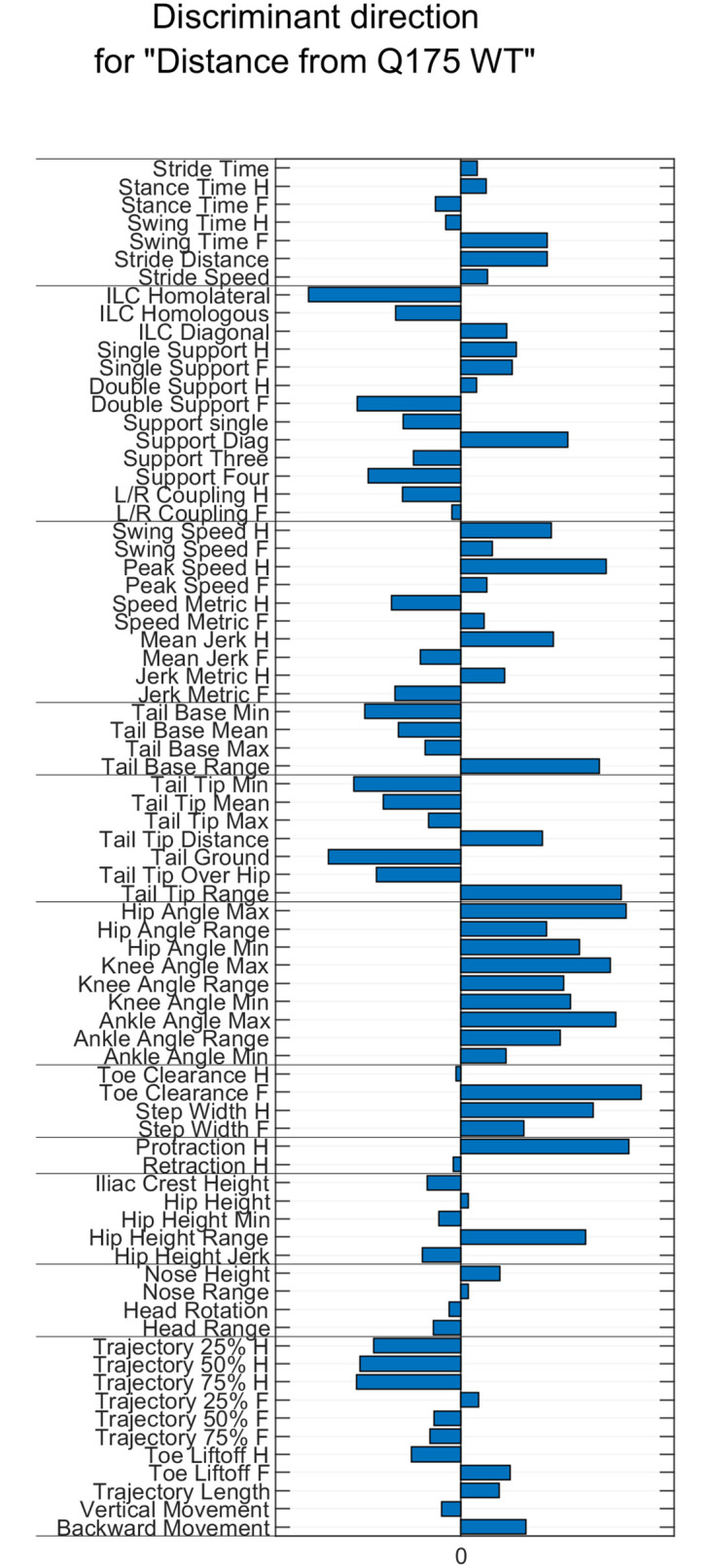
Discriminant vector bar graph of principal component (PC) analysis. The vectors represent unique or enriched features (combination of kinematic variables) in Q175 compared to WT group. In the vector bar graph, the bar length and direction correspond to the weight of individual parameters in the corresponding PC.

**Fig 3:**
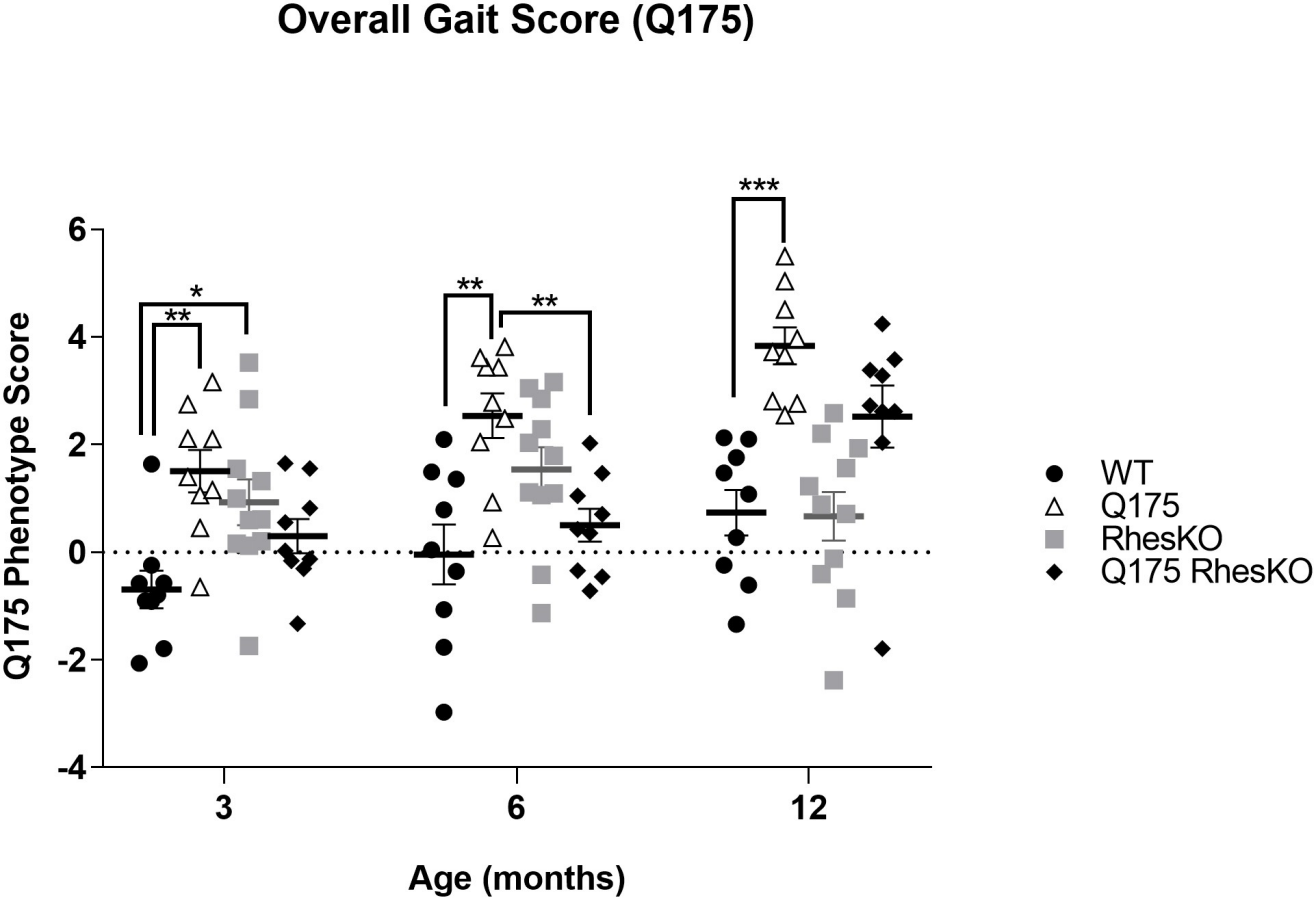
Overall gait score in Q175 female mice. The overall gait score based on differences between Q175 and WT was identified. Scores project normalized parameter data of each mouse onto the discriminant vector; two-way mixed ANOVA followed by Tukey’s test (WT n = 9; RhesKO n = 11; Q175 n = 9; Q175;RhesKO n = 9); Two-way mixed ANOVA followed by Tukey’s test, * p < 0.05, ** p ≤ 0.01, *** p < 0.001.

**Q175 phenotype**. The Q175 mice exhibited deficits in fine motor capabilities as reported earlier [31]. In addition to the overall gait score (Figs 2 and 3) a varimax analysis [31] for the data, is presented in supplementary data (S2 Fig). The clearest Q175 phenotype was observed in parameters such as joint angle ranges, and especially in the parameters describing the degree of within subject variation of the joint angle ranges. Increased variance in these features was observed in Q175 mice compared to WT mice (Figs 2 and 3 and S2 Fig). Related to these changes, back body posture was lowered in the Q175 mice, as indicated by lower tail base height, accompanied with increased hind limb protraction, seen especially at the latest time point of 12 months of age (Figs 2 and 3 and S2 Fig). Both of these findings are in line with the previously reported findings [31]. In addition to these, height of the tail tip was lower, hind limb step width was wider (Figs 2 and 3), and hind limb trajectory shapes were altered in the Q175 mice, compared to WT mice. A few gait features were observed in the Q175 mice in the current study that somewhat differed from those described before [31]. There were no significant differences in the movement speed or the different modes of cadence between the genotypes, both of which were significantly altered in the previous study [31]. However, otherwise the Q175 phenotype was close to similar in both studies.

**Rhes phenotype**. The Rhes KO mice differed significantly in a few features from the WT mice, however significant changes were seen mostly at 3 months of age. Specifically, the overall movement speed of the Rhes KO mice was increased (S2 Fig: PC#1). In addition, the Rhes KO mice exhibited decreased stance time and double support accompanied with increased hind limb toe clearance compared to WT mice (S2 Fig: PC#9). Rhes KO induced changes on the Q175 phenotype were quite sparse, however there were significant effects on a few features. The overall gait score of Q175;Rhes KO mice was recovered towards the WT mice significantly at 6 months of age (Figs 2 and 3). This was mostly due to the effect on excess vertical hip movement, which was evident in Q175 mice and attenuated in Q175;Rhes KO mice (Figs 2 and 3 and S2 Fig: PC#5). However, swing time appeared to be negatively impacted in Q175;Rhes KO mice and hindlimb trajectory and jerkiness changed in the 12 month Q175, and not attenuated in Q175;Rhes KO (S2 Fig: PC#7 and PC#10).

### Brain volumetry

**Fig 4:**
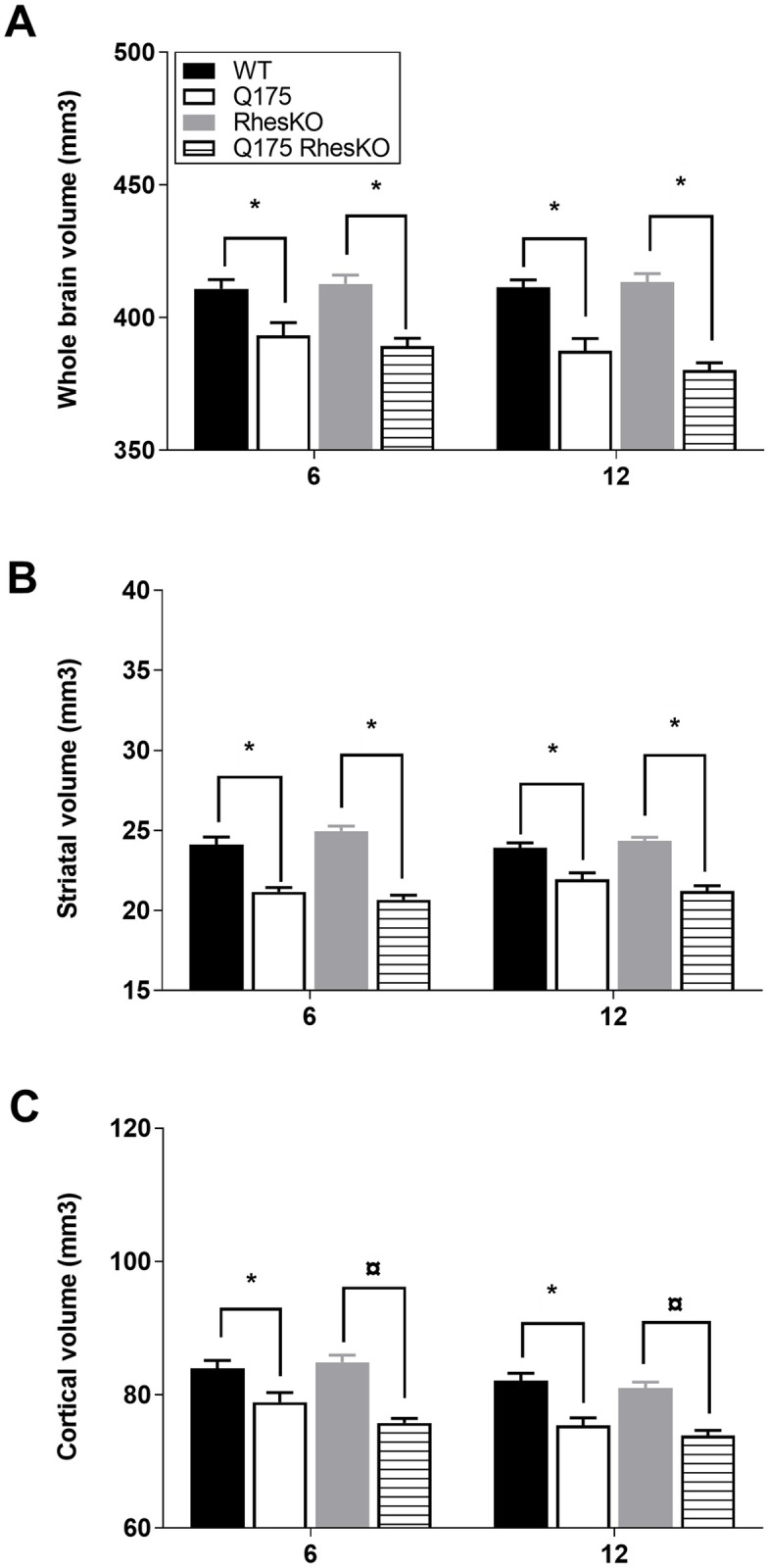
MRI analysis of Q175/Rhes female mice at 6–12 months of age. (A) whole brain volume, (B) striatal volume and (C) cortical volume. Data are presented as mean, + SEM (WT n = 9; RhesKO n = 11; Q175 n = 9; Q175;RhesKO n = 9); Two-way mixed ANODE followed by Tukey’s test, * p < 0.05, Q175 vs. WT andQ175;RhesKO vs. RhesKO.

RhesKO had no significant effects on brain volume in Q175 mice. Simple main effects analysis showed a significant difference for Q175 (two-way mixed ANODE, p = 0.001), but not for Rhes genotype (p = 0.578). Q175 mice had decreased whole brain volumes compared to WT mice at 6 and 12 months of age (p = 0.041, p < 0.0004, respectively; Fig 4A). Q175;RhesKO mice had decreased whole brain volumes compared to RhesKO mice at 6 and 12 months of age (p < 0.0003, p < 0.0001, respectively). There was no difference in whole brain volume between WT and RhesKO at 6 or 12 months of age (p > 0.05). RhesKO had no impact on whole brain volume atrophy in the Q175 (Q175 vs. Q175;RhesKO p > 0.05). There was a statistically significant interaction between the effects of Q175 and Rhes genotypes on striatal volumes (two-way ANOVA, p = 0.032), and a significant interaction between Q175 genotype and age (two-way ANOVA, p = 0.001; Fig 4B). Simple main effects analysis showed a significant difference for the Q175 (p < 0.0001), but not for Rhes genotype (p = 0.066). Q175 mice had decreased striatal volume compared to WT mice at 6 and 12 months of age (p < 0.0001, p < 0.0001, respectively). Q175;RhesKO mice had decreased striatal volumes compared to RhesKO mice at 6 and 12 months of age (p < 0.0001, p < 0.0001, respectively). There was no difference in striatal volume between WT and RhesKO at 6 or 12 months of age (p > 0.05). RhesKO had no impact on striatal volume atrophy in the Q175 (Q175 vs. Q175;RhesKO p > 0.05; Fig 4B). There was no statistically significant interaction between the effects of Q175 and Rhes genotypes on cortical volumes (two-way mixed ANODE, p = 0.235; Fig 4C). Simple main effects analysis showed a strong significant difference for Q175 (p < 0.0001), but not for Rhes genotype (p = 0.981). Q175 mice had smaller cortical volume, compared to WT mice, and age had an effect, cortical volume decreased from 6 to 12 months of age (p = 0.003, p = 0.005, respectively). Also, Q175;RhesKO mice had decreased cortical volumes compared to RhesKO mice at 6 and 12 months of age (p < 0.0001, p < 0.0001, respectively). There was no difference in cortical volume between WT and RhesKO at 6 or 12 months of age (p > 0.05). RhesKO had no impact on cortical volume atrophy in the Q175 (Q175 vs. Q175;RhesKO p > 0.05; Fig 4C).

### MR spectroscopy

**Fig 5:**
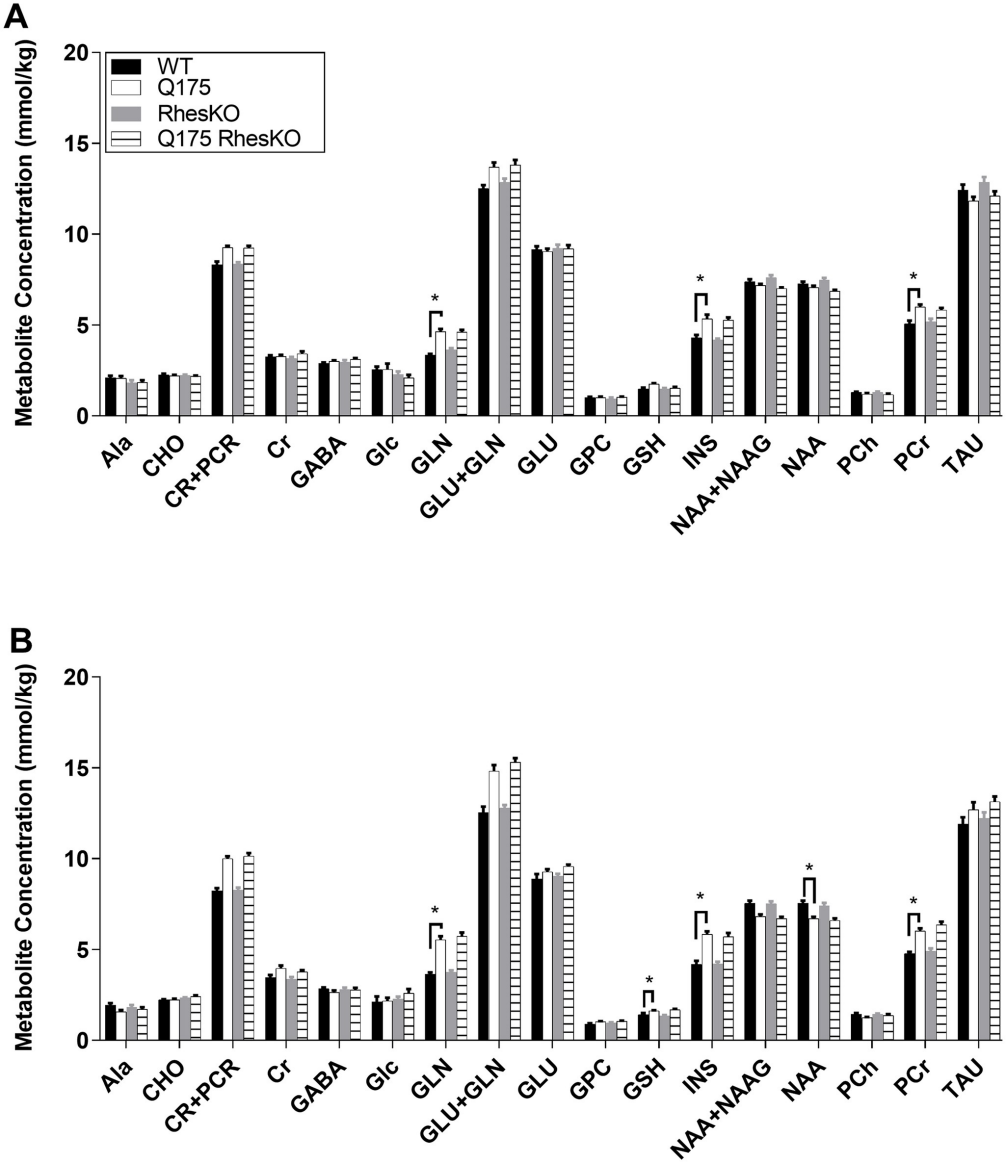
MR Spectroscopy analysis of Q175/Rhes female mice, (A) at 6 months of age and (B) at 12 months of age, presenting measured striatal metabolite concentrations from: Ala, alanine; CHO, choline; CR+PCR, creatine+phosphocreatine; CR, creatine; GABA, gamma-aminobutyric acid; Glc, glucose; GLN, glutamine; GLU+GLN, glutamate+glutamine; GLU, glutamate; GPC, glycerophosphocholine; GSH, glutathione; INS, myo-inositol; NAA+NAAG, N-acetylaspartate+N-acetylaspartylglutamate; NAA, N-acetylaspartate; PCh, phosphocholine; PCr, phosphocreatine; TAU, taurine;. Data are presented as mean, + SEM (WT n = 9; RhesKO n = 11; Q175 n = 9; Q175;RhesKO n = 9); Two-way mixed ANODE followed by Tukey’s test, * p < 0.05.

The striatal metabolic profile of Q175 mice was very similar to what was previously reported [37]. For both phosphocreatine and glutamine, there was a strong significant interaction between Q175 genotype and age (two-way ANOVA, p = 0.004 and p < 0.0001, respectively), Q175 mice had increased phosphocreatine and glutamine levels at 6 and 12 months, compared to WT mice (p < 0.05; Fig 5). For glutathione concentrations simple main effects showed significant differences for Q175 genotype (p = 0.012) and age (p = 0.013), the glutathione levels were increased in Q175 mice at 12 months of age compared to WT mice (p < 0.05; Fig 5). There was a statistically significant interaction with Q175 genotype and age in myo-inositol and N-acetyl-aspartate (NAA) concentrations (two-way ANOVA, p = 0.024 and p = 0.014, respectively; Fig 5). Q175 mice had increased myo-inositol concentrations both at 6 and 12 months of age (p < 0.001; Fig 5), and significantly decreased NAA concentrations at 12 months of age (p < 0.05; Fig 5), compared to WT mice. Q175;RhesKO did not exhibit altered metabolic profiles, compared to WT or Q175.

### RhesKO did not modulate DARPP32, autophagy or mHTT in the striatum

**Fig 6:**
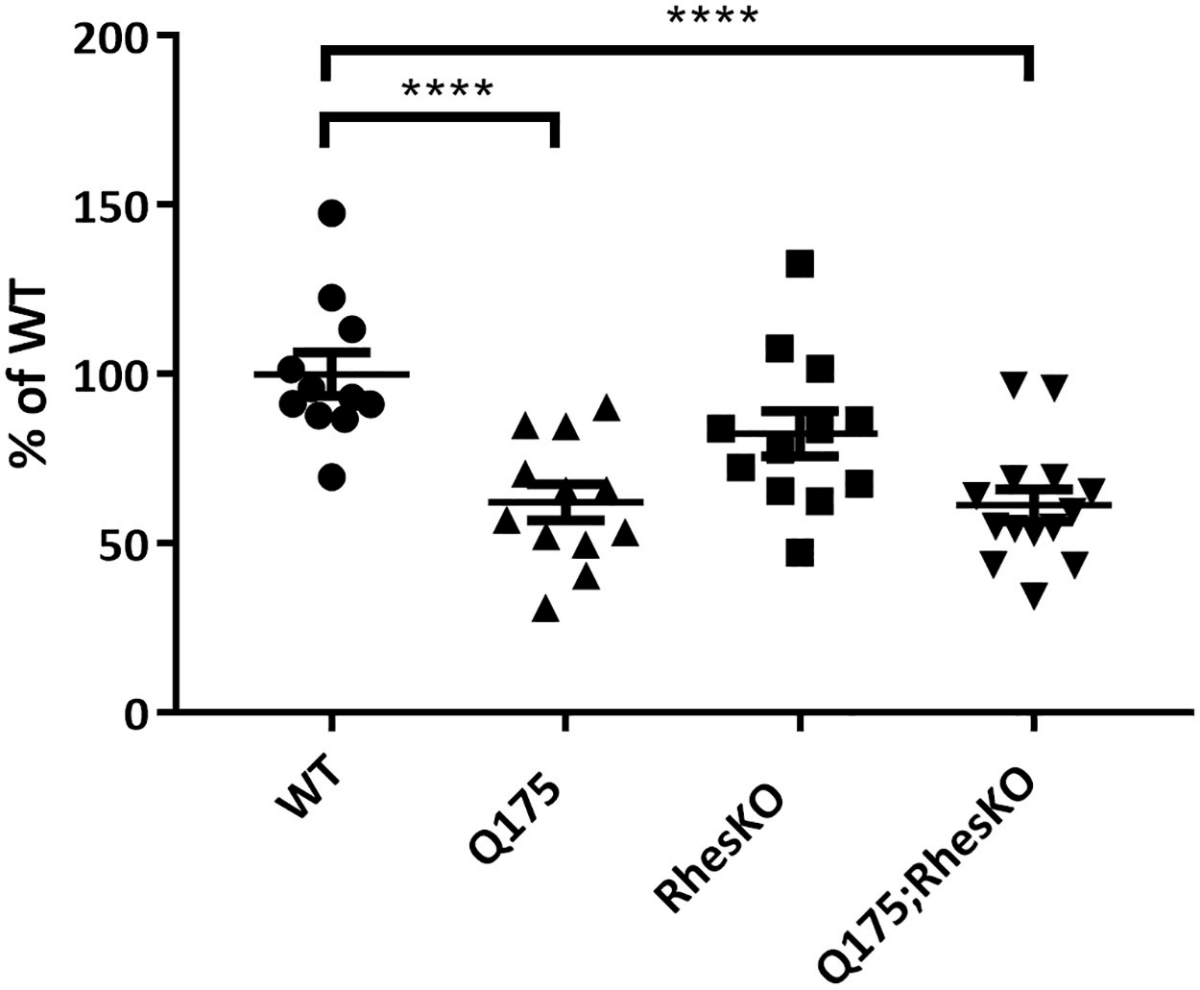
RhesKO did not restore DARPP32 striatal deficit in mixed gender Q175 mice. Quantitation of western blots of 5 month striatal samples from male and female mice probed for DARPP32. For each sample, protein level was normalized to in-lane housekeeping protein (vinculin) and presented as percent of WT. Data presented as mean ± SEM (WT n = 6 females, 5 males; RhesKO n = 5 females, 6 males; Q175 n = 6 females, 6 males; Q175;RhesKO n = 6 females, 8 males); One way ANOVA with Bonferroni multiple comparison test; *p<0.05, ****p<0.0001).

**Fig 7:**
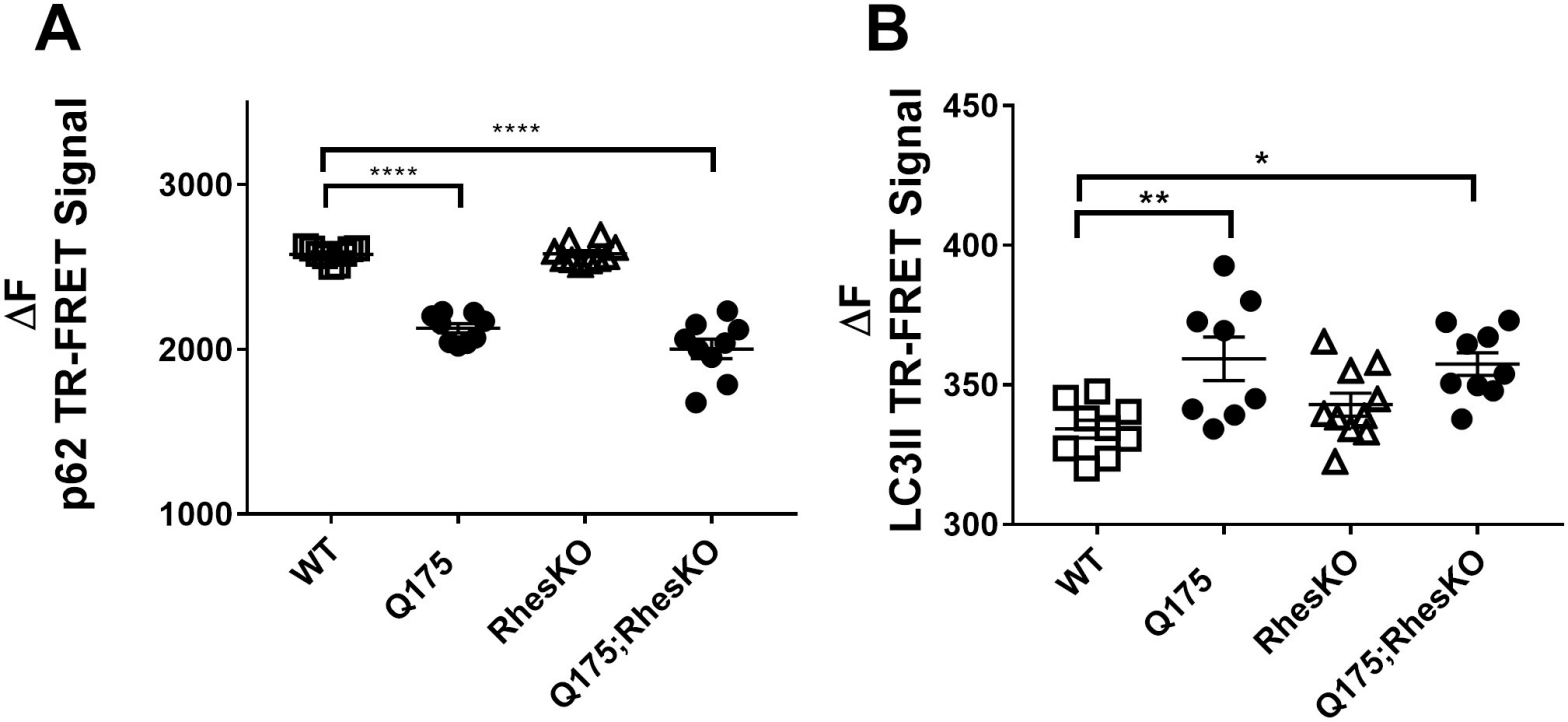
RhesKO did not modulate autophagy in female Q175 mice. TR-FRET for p62 (A) and lipidated LC3II (B) was performed on 12 month female mouse striatum. Data are expressed as mean ± SEM (WT n = 9; RhesKO n = 11; Q175 n = 9); Q175;RhesKO n = 9; One-way ANOVA statistical analysis: * p <0.05, ** p < 0.01, **** p < 0.0001).

**Fig 8:**
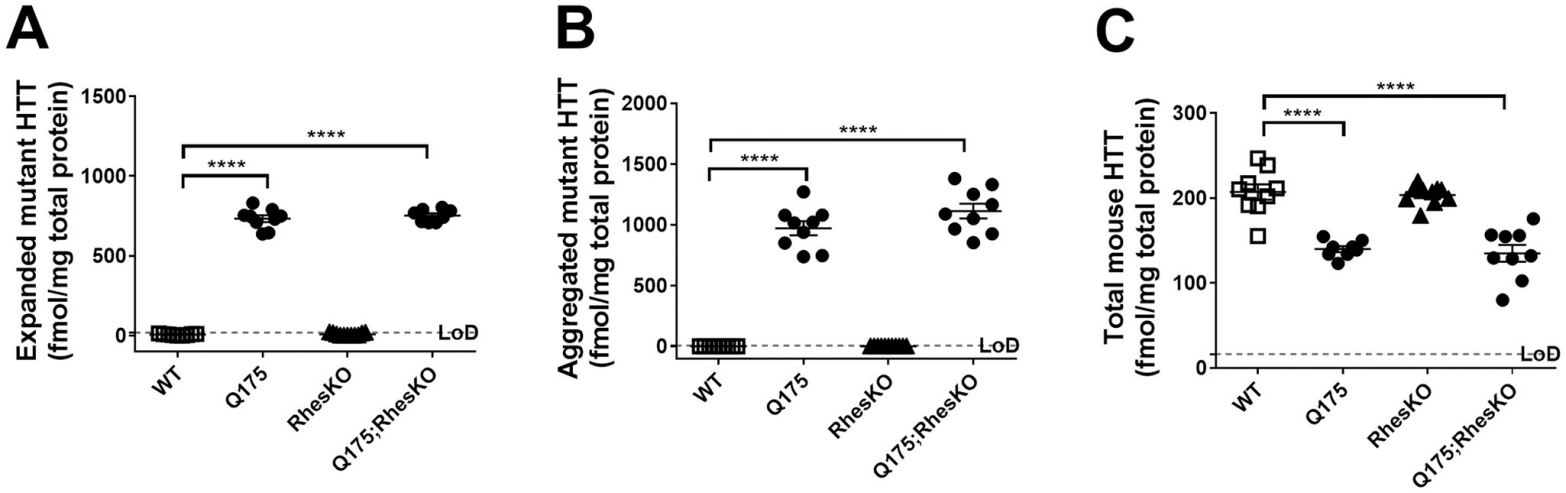
RhesKO did not modulate HTT levels in female Q175. MSD for soluble, aggregated and total endogenous mouse HTT was performed on 12 month striatum. Data are expressed as mean ± SEM (WT n = 9; RhesKO n = 11; Q175 n = 9; Q175;RhesKO n = 9); One-way ANOVA statistical analysis: * p <0.05, ** p < 0.01, **** p < 0.0001).

As expected, there was a significant reduction in DARPP32 protein in the 5 month striatum of Q175, compared to WT (One-way ANOVA with Bonferroni multiple comparisons tests were performed; ****p<0.0001). There was no restoration of DARPP32 protein in the Q175;RhesKO, compared to Q175 (p>0.05; Fig 6). Neuronal loss has not been reported in this model until 2 years, suggesting loss of DARPP32 expression levels in neurons [38,39]. Autophagy appeared to be changed in the 12 month striatum of Q175 mice, compared to WT, as p62 protein was down and lipidated LC3II was increased (p<0.01 and p<0.001, respectively; One-way ANOVA). RhesKO had no impact on the p62 and LC3II changes observed in the Q175 (Q175 vs. Q175;RhesKO; Fig 7). There was an increase in soluble and aggregated mHTT and a decrease in endogenous mouse HTT in the 12 month Q175 striatum, compared to WT (n = 9–10; p<0.0001). RhesKO had no impact on HTT or mHTT levels in the Q175;RhesKO compared to Q175 (Fig 8).

### Rhes does not modulate basal mTOR signaling in the Q175 mouse model

**Fig 9:**
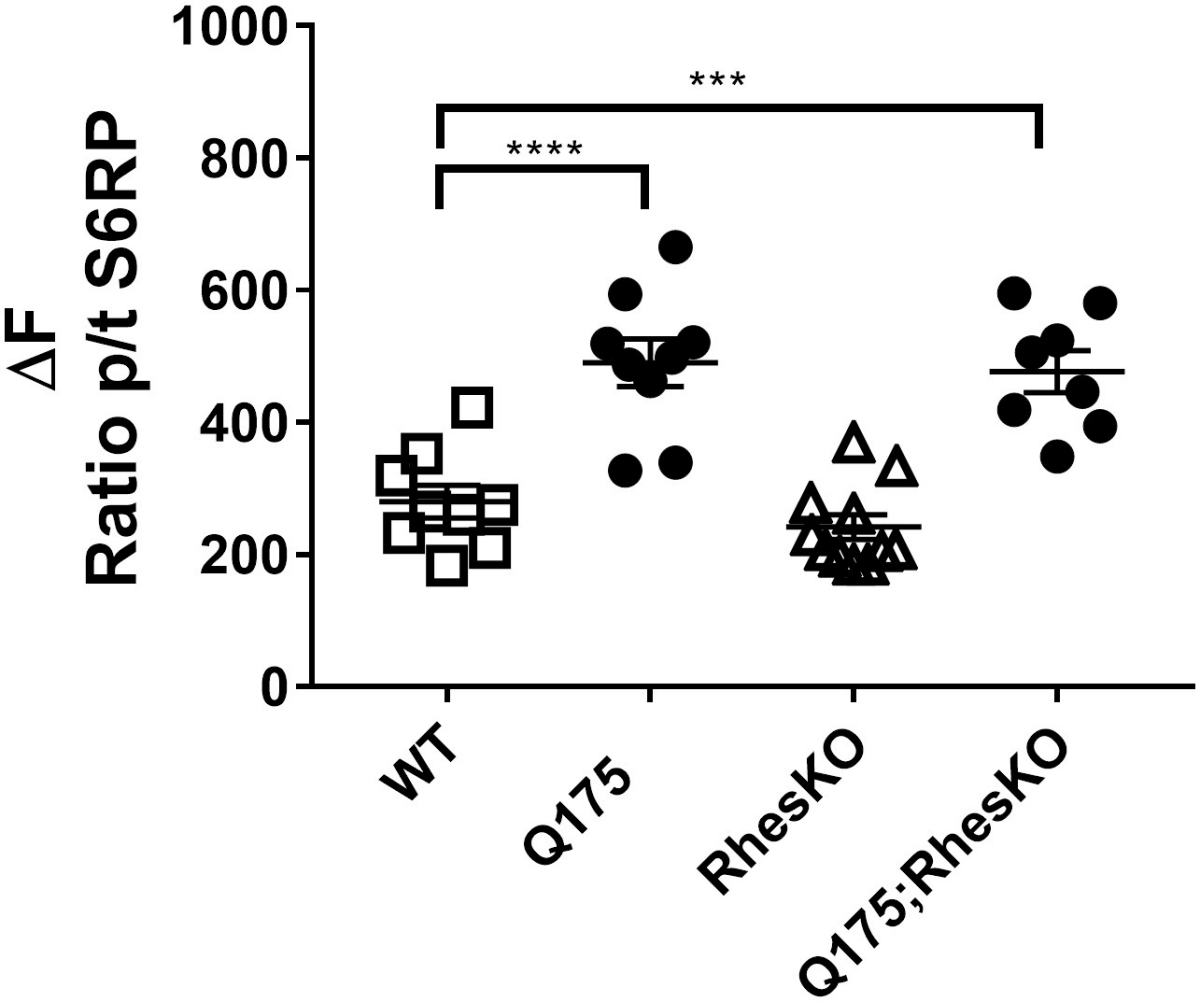
RhesKO did not modulate mTOR signaling in female Q175. MSD analysis of p-S6^**S235/236**^ normalized by S6 in the 12 month striatum. Data are expressed as mean ± SEM (WT n = 9; RhesKO n = 11; Q175 n = 9; Q175;RhesKO n = 9); One-way ANOVA statistical analysis: **** p < 0.0001).

Several phosphor-proteins in the mTOR signaling pathway were examined to determine if mTOR signaling is aberrant in the Q175 striatum and whether Rhes modulates mTOR signaling in the striatum. P-S6^**S235/236**^, p-mTOR^**S2448**^/total mTOR and p-4EBP1^**S65**^/ total eBP1 were examined by western blotting or Luminex in the striatum of 5 and 12 month old mice. P-S6K^**T389**^ was examined by Luminex at 12 months only. There were no differences in mTOR signaling between Q175 and WT, nor was there any difference between WT and RhesKO or Q175 and Q175;RhesKO (S3 and S4 Figs). In contrast, p-S6^**S235/236**^ was measured by MSD assay in the 12 month striatum and was significantly increased in Q175, compared to WT (p<0.0001). Genetic RhesKO had no impact on p-S6^**S235/236**^, when measured by MSD (Fig 9).

### Rheb may have a compensatory role in Q175 and RhesKO models

**Fig 10:**
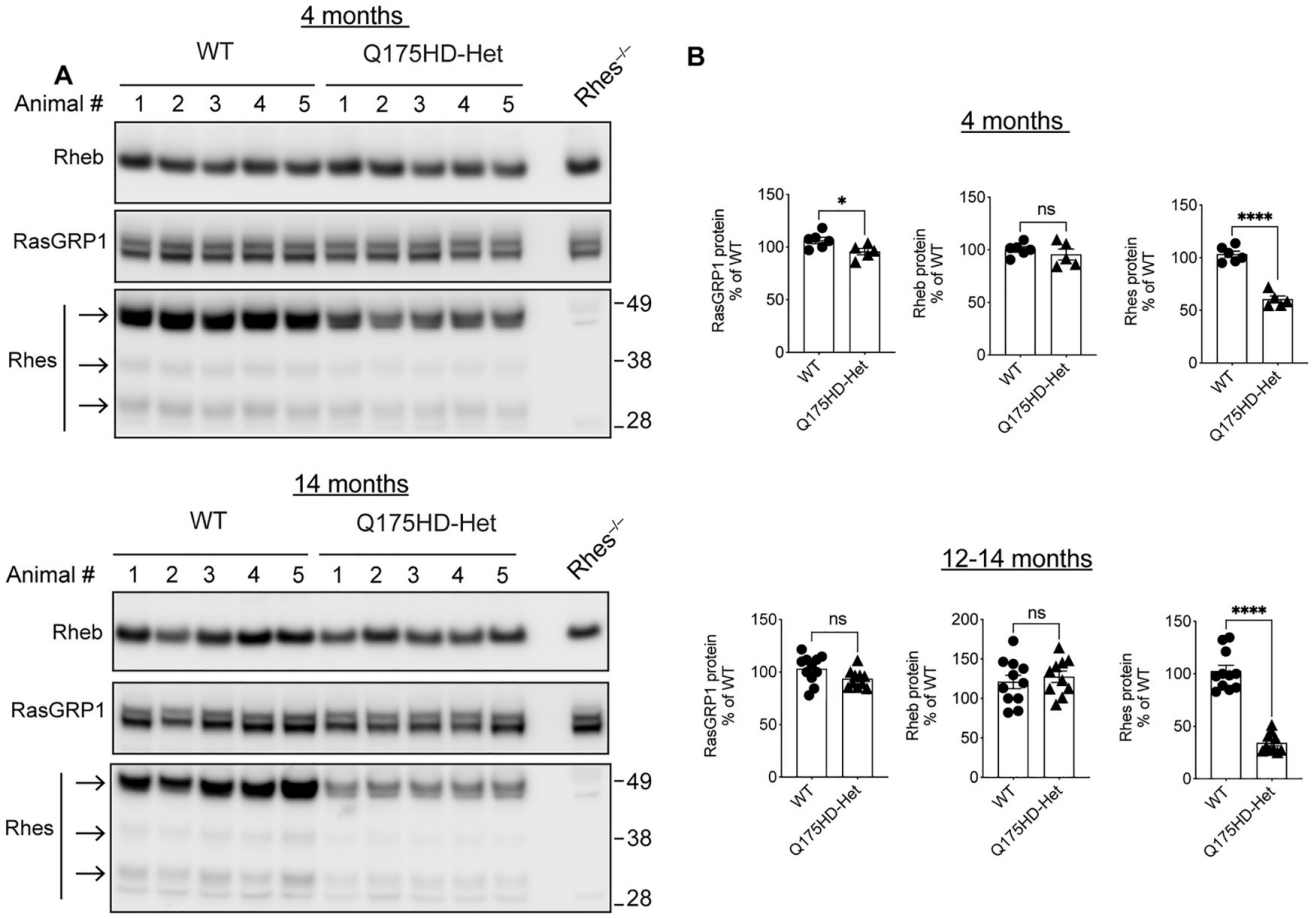
Rhes is downregulated in the striatum of mixed gender Q175. Western blot of striatum from 4M and 14M WT and Q175 mice (A). Quantification of RasGRP1, Rheb and Rhes protein. Data are expressed as mean ± SEM (4M: WT n = 6 male, Q175 n = 5 male and 12-14M: WT n = 9 male, n = 2 female, Q175 n = 9 male, n = 1 female *p<0.05, ****p<0.0001, Student’s t-test.

Because Rhes exists in modified forms in the striatum [30,40,41], we determined Rhes levels in the Q175 striatum by western blot. All three different forms of Rhes protein were analyzed together and found to be significantly reduced in the Q175 striatum at 4 months of age (n = 5–6; p<0.0001) and 12–14 months of age (n = 10–11; p<0.0001). RasGRP1, a guanine nucleotide exchange factors (GEF) implicated in the activation of striatal mTORC1 signaling [42,43], was slightly decreased at 4 months (p<0.05), but not at 12–14 months. Rheb protein, a major activator of mTORC1 [44], was not altered in the striatum at both ages examined (Fig 10). While Rhes is downregulated in the Q175 mouse model, other mTORC1 regulators remain unaltered, which may still promote mTORC1 activation in the striatum.

## Discussion

Using diverse cell and mouse HD models, conflicting reports describe the modulation of Rhes as a therapeutic strategy. Rhes expression exacerbates cell death in the presence of mHTT in HEK293 and striatal-cell models [11,17,19]. In PC12 cell, hESC-derived neuron, or primary striatal neuron HD models, Rhes depletion prevents cell death [11,16,18]. Previous studies report both protective or worsening behavioral performance in HD mouse models with Rhes modulation [9,10,25–27]. In contrast to published studies using mHTT fragment and transgenic mouse models, our study used the full-length Q175 knock-in mouse model. Here we performed a motor kinematic analysis measuring numerous features; in agreement with previous studies using the Q175 mouse model, females exhibited impairments in fine motor skills, compared to WT [37] and these impairments were attenuated with genetic RhesKO at 6 months of age, but not 3 or 12 months of age. Specifically, PC#5, representing excess vertical hip movement, identified a phenotype in the Q175 that was ameliorated with RhesKO. In contrast, PC#7 identified that swing time was not changed in Q175 at 12 months of age, but was reduced in RhesKO, compared to all other groups, suggesting a detrimental impact of RhesKO. The behavioral analysis should be considered with care since genetic RhesKO alone impacts fine motor behavior. We acknowledge that our study did not include both genders, therefore this should be taken into consideration as previous work suggests that Rhes may have gender effects on striatal-dependent behaviors [27,45–47]. Enlargement of lateral ventricles in the N171-82Q HD mouse model was reported to be attenuated by genetic RhesKO [27]. In contrast, no changes in brain weight or area were reported in the R6/1 mouse model with RhesKO, however, it is notable that RhesKO decreased brain weight and area, compared to WT [26]. While Q175 brains have lower total brain and striatal volume, as measured by MRI, at 6 and 12 months, genetic RhesKO had no effect using this sensitive measurement. Furthermore, we report changes in the striatal metabolic profile in Q175 mice as measured by MR spectroscopy, which was not affected by RhesKO. Overall, genetic RhesKO did not robustly affect behavioral or neurodegenerative deficits in a full-length HTT mouse model, Q175.

It is plausible that Rhes needs to be modulated in the adult brain to avoid compensatory responses that would stem from genetic RhesKO, and previous studies have addressed this question using viral approaches. AAV- microRNA-based reduction of Rhes mRNA in the striatum of N171-82Q mice resulted in no effect on rotarod performance, whereas in BACHD mice the same approach resulted in striatal atrophy and hypoactivity in the activity chamber [9]. In contrast, overexpression of Rhes using AAV-Rhes in the N171-82Q mouse striatum resulted in improvements in rotarod performance, but the mice were still significantly impaired compared to WT mice [10]. However, in that study Rhes was flag-tagged at the C-terminal end that may affect farnesylation and mislocalize Rhes to the nucleus [11], and a Rhes overexpression effect could not be excluded since WT control mice were not included [10]. In an additional study, adenovirus-mediated expression of untagged Rhes in the cerebellum of N171-82Q mice or expression in the striatum of Q150;RhesKO mice worsened motor deficits [27]. It is also possible that since Rhes expression is already down in the Q175 mouse [8–10] (HDinHD: www.hdinhd.org; Fig 10), additional lowering of Rhes may not be expected to further impact the phenotypes of this mouse model.

None of the previous studies comprehensively examined multiple endpoints within either a single study or cohort of mice in a well-characterized knock-in mouse model [9,10,26,27]. Here, we comprehensively investigated the effect of genetic RhesKO on multiple readouts within each individual female Q175 mouse, examining behavior, volumetry, metabolic profiles, autophagy, mHTT levels and mTOR signaling. We also examined mTOR signaling and DARPP32 in a separate group of mixed gender mice. Our findings show that genetic RhesKO did not affect Q175 HD phenotypes.

Since mHTT can promote mTORC1 activation via Rheb in cell culture and Rhes activates striatal mTORC1 signaling by L-DOPA in the striatum [20,21,23], we examined the effect of Rhes on mTOR signaling in Q175 mouse striatum. However, it is unclear whether the mTOR pathway is dysregulated in HD [10,23,48–51]. mTOR activity is commonly measured by phosphorylation of mTOR substrates and the data is often variable, which may explain why there is disagreement between studies. Furthermore, mTOR signaling is dynamic [52,53], which makes it difficult to come to a robust conclusion about mTOR signaling in HD. Even within our study we found no change in mTOR signaling between Q175 and WT striatum when we measured pS6^**S235/236**^, p-mTOR^**S2448**^ and p-4EBP1^**S65**^/total with western blots, while we detected an increase in mTOR signaling in the Q175 striatum when measured by pS6^**S235/236**^ MSD. In the same model, a significant upregulation in striatal mTOR1 signaling, as measured by p-mTOR^**S2448**^ and pS6K westerns was reported [54]. These results suggest that mTORC1 signaling in the Q175 may be highly dynamic. Nonetheless, with all methods used we found that genetic RhesKO did not alter mTOR signaling in WT or Q175 striatum. Although we expected that Rhes might influence mTOR signaling, Rhes levels were diminished in the Q175 striatum [8–10] (HDinHD: www.hdinhd.org; Fig 10) suggesting that Rhes may not adequately control mTOR in the Q175 striatum under basal conditions. Rheb, a regulators of mTORC1, was not altered in the Q175 striatum and may have a compensatory role in regulating mTORC1 signaling.

While mTOR signaling can regulate autophagy [55,56], autophagy can also be stimulated by mTOR-independent pathways [57,58]. In cultured cells, Rhes can promote autophagy in association with Beclin1 and mitophagy by interaction with Nix [59,60]. We detected an increase in autophagy, as measured by LCII and p62 TR-FRET, in the Q175 striatum at 12 months; previous reports suggest no changes to subtle changes in autophagy in HD mouse models [50,61–65]. We did not detect any changes in autophagy or mHTT protein in the genetic RhesKO on the WT or Q175 background. Ours is the first study to examine the impact of Rhes on HTT protein levels *in vivo*, and we found no effect using our methods.

An *in vivo* role for SUMOylation has been suggested in HD [66]. Rhes binds and promotes SUMOylation of mHTT in culture, which changes the aggregation and toxicity of mHTT [11,12]. We did not examine HTT SUMOylation in this study since the rest of our readouts were negative.

This study aimed to examine multiple readouts within the same mice with genetic RhesKO;Q175, and did not find robust amelioration or exacerbation in the numerous endpoints measured.

## Supporting information

S1 Fig Expression of Rhes. (A) Amplification curves of WT and RhesKO striatal cDNA. (B) ATP5B shows cDNA was successfully prepared from RNA. (C) Western blot from RhesKO and Q175;RhesKO striatum.
(TIF)

S2 Fig Fine motor and gait changes using PCA. Varimax Principal component (PC) scores PC#1–10 are illustrated. The corresponding PCs (eigenvectors) are shown to the right and the percentage describes the proportion of variation in the whole data set that each PC comprises. Data are presented as mean ± SEM (WT n = 9; RhesKO n = 11; Q175 n = 9; Q175;RhesKO n = 9); Two-way mixed ANOVA followed by Tukey’s test, * p < 0.05, RhesKO vs. WT; # p < 0.05, Q175 vs. WT; § p < 0.05, Q175;RhesKO vs. Q175.
(TIF)

S3 Fig Rhes did not modulate mTOR signaling at 5 months in mixed gender Q175 mice. Quantification of western blots of 5 month striatal samples for pAkt^**S473**^/Akt (A), p-mTOR^**S2448**^/mTOR (B) and p-4EBP1^**S65**^/4EBP1 (C). For each sample, protein level was normalized to in-lane housekeeping protein (β-tubulin) and presented as percent of WT ± SEM. There were no changes in mTOR signaling in the Q175 striatum, compared to WT and RhesKO had no impact on mTOR signaling (WT n = 6 females, 5 males; RhesKO n = 5 females, 6 males; Q175 n = 6 females, 6 males; Q175;RhesKO n = 6 females, 8 males).
(TIF)

S4 Fig Rhes did not modulate mTOR signaling at 12 months in mixed gender Q175 mice. Striatal samples were examined by Luminex and bar graphs are represented for pAkt^S473^/Akt (A), p-mTOR^S2448^/mTOR (B), p-4EBP1^S235^/4EBP1 (C) and p-S6K^T389^/S6K (D), all normalized to β-tubulin and presented as percent of WT ± SEM. There were no changes in mTOR signaling in the Q175 striatum, compared to WT, and RhesKO had no impact on mTOR signaling (WT n = 6 females, 5 males; RhesKO n = 5 females, 6 males; Q175 n = 6 females, 6 males; Q175;RhesKO n = 6 females, 8 males).
(TIF)

S1 File (PDF)

S1 Raw images (TIF)
