# Supplementary figures and images for: Global Rhes knockout in the Q175 Huntington’s disease mouse model
Source: PLoS One. 2021 Oct 14;16(10):e0258486. doi: 10.1371/journal.pone.0258486 (PMC8516231; doi:10.1371/journal.pone.0258486)

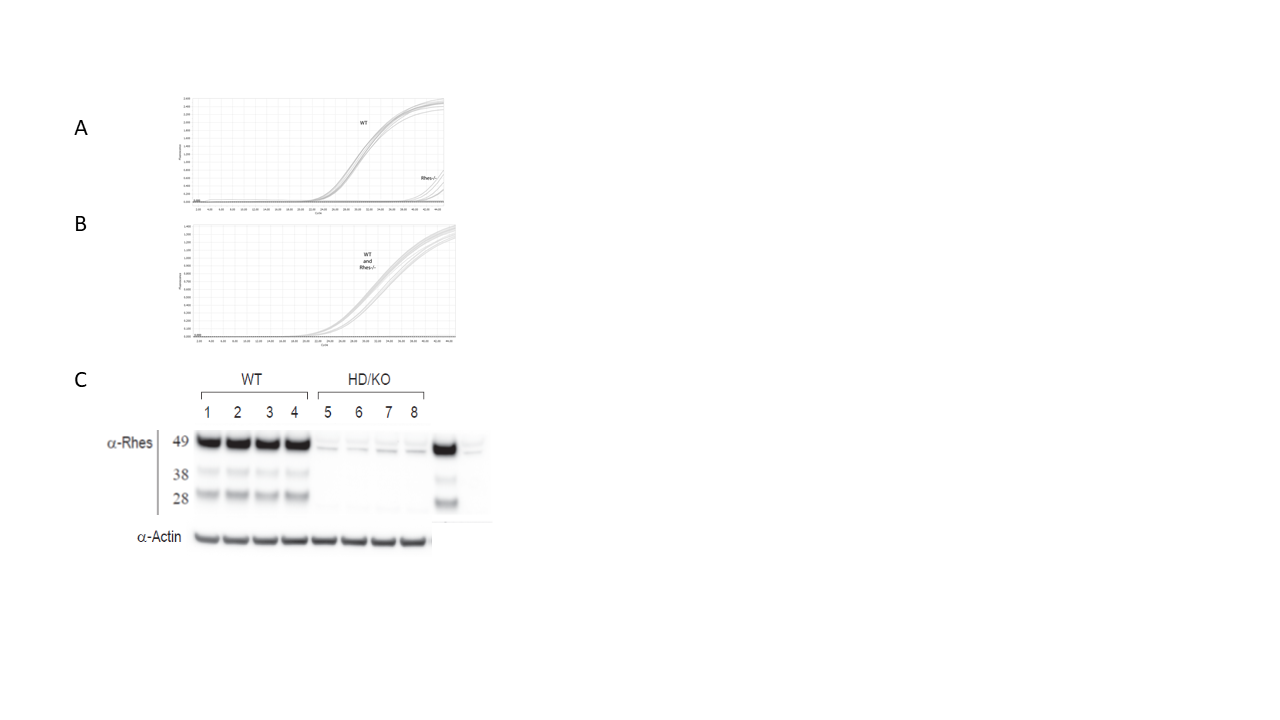

Supplement: S1 Fig — (A) Amplification curves of WT and RhesKO striatal cDNA. (B) ATP5B shows cDNA was successfully prepared from RNA. (C) Western blot from RhesKO and Q175;RhesKO striatum. (TIF) [file pone.0258486.s001.tif]

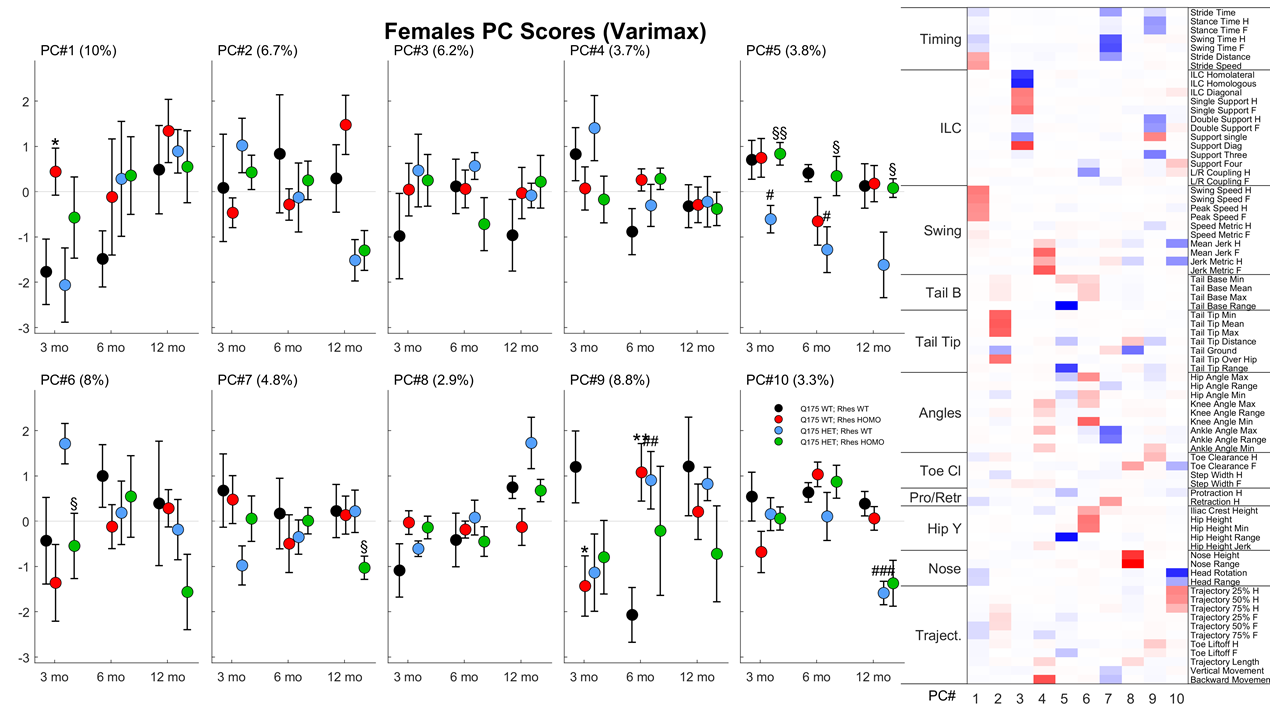

Supplement: S2 Fig — Varimax Principal component (PC) scores PC#1–10 are illustrated. The corresponding PCs (eigenvectors) are shown to the right and the percentage describes the proportion of variation in the whole data set that each PC comprises. Data are presented as mean ± SEM (WT n = 9; RhesKO n = 11; Q175 n = 9; Q175;RhesKO n = 9); Two-way mixed ANOVA followed by Tukey’s test, * p < 0.05, RhesKO vs. WT; # p < 0.05, Q175 vs. WT; § p < 0.05, Q175;RhesKO vs. Q175. (TIF) [file pone.0258486.s002.tif]

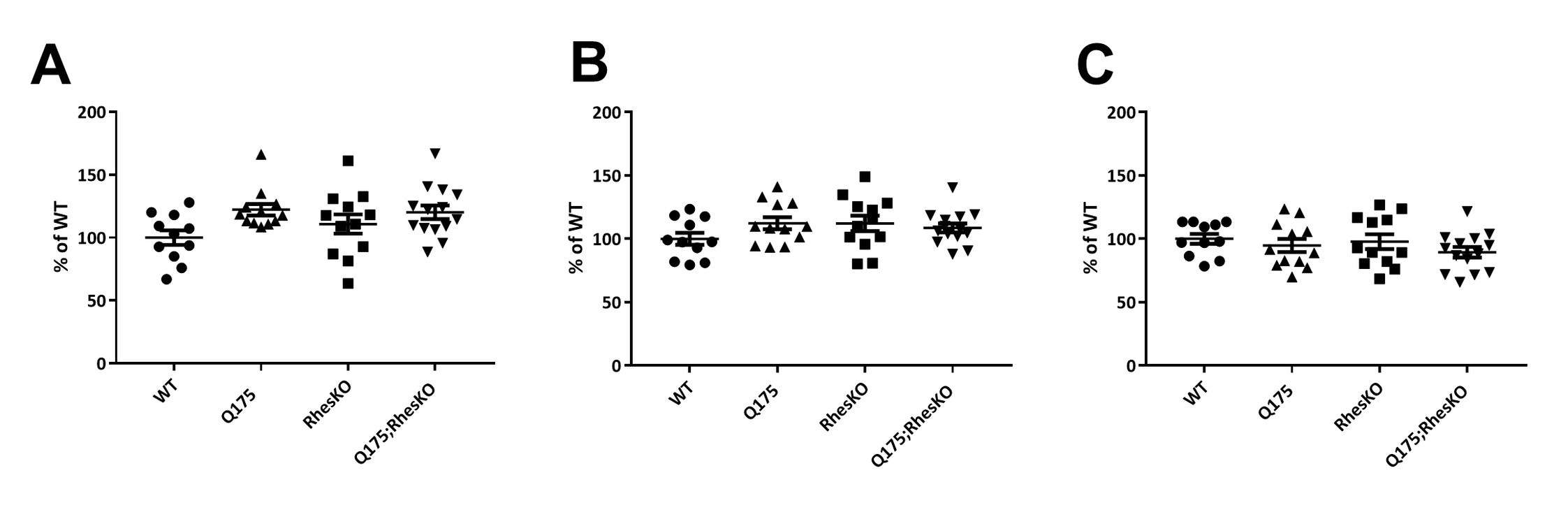

Supplement: S3 Fig — Quantification of western blots of 5 month striatal samples for pAktS473/Akt (A), p-mTORS2448/mTOR (B) and p-4EBP1S65/4EBP1 (C). For each sample, protein level was normalized to in-lane housekeeping protein (β-tubulin) and presented as percent of WT ± SEM. There were no changes in mTOR signaling in the Q175 striatum, compared to WT and RhesKO had no impact on mTOR signaling (WT n = 6 females, 5 males; RhesKO n = 5 females, 6 males; Q175 n = 6 females, 6 males; Q175;RhesKO n = 6 females, 8 males). (TIF) [file pone.0258486.s003.tif]

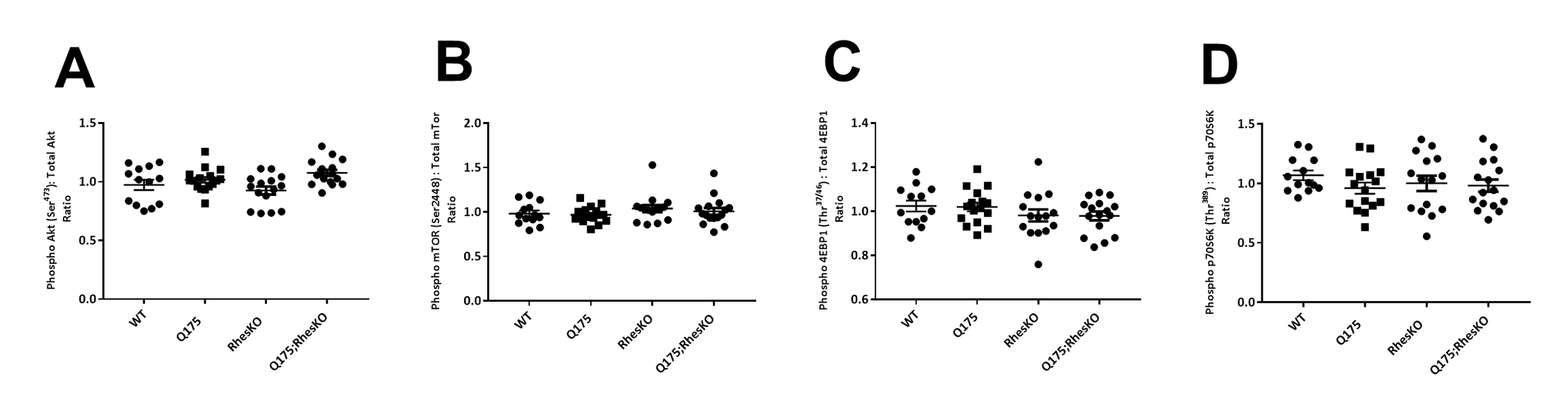

Supplement: S4 Fig — Striatal samples were examined by Luminex and bar graphs are represented for pAktS473/Akt (A), p-mTORS2448/mTOR (B), p-4EBP1S235/4EBP1 (C) and p-S6KT389/S6K (D), all normalized to β-tubulin and presented as percent of WT ± SEM. There were no changes in mTOR signaling in the Q175 striatum, compared to WT, and RhesKO had no impact on mTOR signaling (WT n = 6 females, 5 males; RhesKO n = 5 females, 6 males; Q175 n = 6 females, 6 males; Q175;RhesKO n = 6 females, 8 males). (TIF) [file pone.0258486.s004.tif]

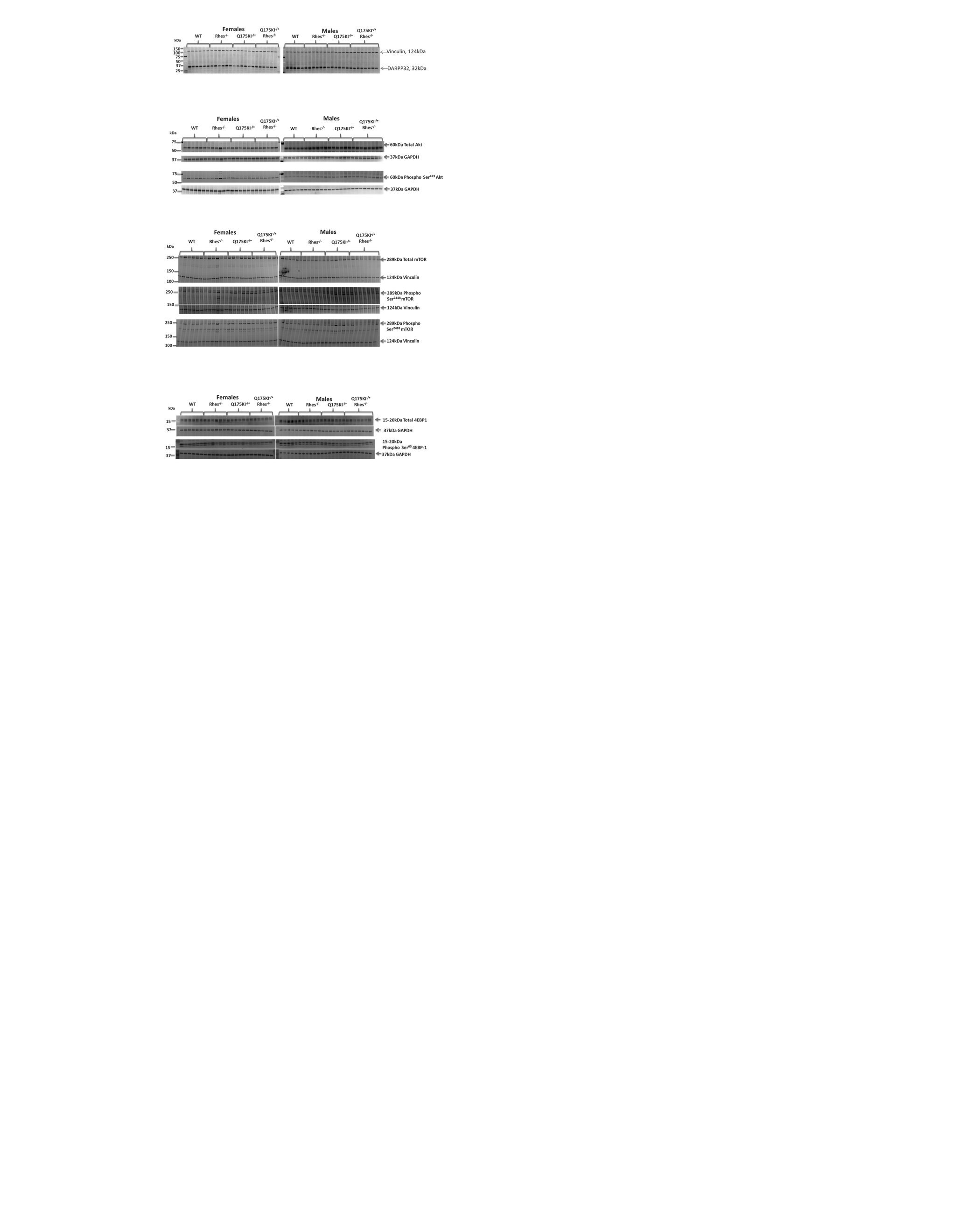

Supplement: S1 Raw images — (TIF) [file pone.0258486.s006.tif]
